# Lactucarium Syrup and Paste

**Published:** 1889-06

**Authors:** 


LACTUCARIUM SYRUP AND PASTE of H. Aubergier, Honorary Dean of the Faculty of Sciences; President of the Academy of Sciences, etc., Paris. "The preparations of Lactucarium have been approved by the *Academie of Medicine*, and their formulæ have been made officinal. And it is claimed that "*Aubergier's Syrup and Paste*" have never been publicly advertised in France or the U. S ; they owe their reputation wholely to unqualified approval of the Medical Profession.

The editors of the RECORD have been kindly furnished with a beautiful sample of the "Syrup." It is claimed that this preparation has endowed the healing art with a medicament as sedative and calmative as opium without having its narcotic disadvantages. It is suited to all cases where an anodyne is indicated, especially in bronchitis, and as an adjuvant to cough mixtures in all lung and bronchial irritations. It is also well adapted to cases of insomnia, to nervous palpitations of the heart, and to intestinal neuralgias. For the sample sent us we are indebted to the accommodating and enterprising house of E. Fougera & Co., New York.

